# A role of the SAM domain in EphA2 receptor activation

**DOI:** 10.1038/srep45084

**Published:** 2017-03-24

**Authors:** Xiaojun Shi, Vera Hapiak, Ji Zheng, Jeannine Muller-Greven, Deanna Bowman, Ryan Lingerak, Matthias Buck, Bing-Cheng Wang, Adam W. Smith

**Affiliations:** 1 Department of Chemistry, University of Akron, Akron, OH, 44325, USA; 2 Departments of Physiology and Biophysics, Case Western Reserve University, Cleveland, OH, 44106, USA; 3 Pharmacology, Case Western Reserve University, Cleveland, OH, 44106, USA; 4 Rammelkamp Center for Research, MetroHealth Medical Center, Cleveland, OH, 44109, USA; 5 Department of Biology, University of Akron, Akron, OH, 44325, USA; 6 Department of Neurosciences, Case Western Reserve University, Cleveland, Ohio, 44106, USA.

## Abstract

Among the 20 subfamilies of protein receptor tyrosine kinases (RTKs), Eph receptors are unique in possessing a sterile alpha motif (SAM domain) at their C-terminal ends. However, the functions of SAM domains in Eph receptors remain elusive. Here we report on a combined cell biology and quantitative fluorescence study to investigate the role of the SAM domain in EphA2 function. We observed elevated tyrosine autophosphorylation levels upon deletion of the EphA2 SAM domain (EphA2ΔS) in DU145 and PC3 prostate cancer cells and a skin tumor cell line derived from EphA1/A2 knockout mice. These results suggest that SAM domain deletion induced constitutive activation of EphA2 kinase activity. In order to explain these effects, we applied fluorescence correlation spectroscopy to investigate the lateral molecular organization of EphA2. Our results indicate that SAM domain deletion (EphA2ΔS-GFP) increases oligomerization compared to the full length receptor (EphA2FL-GFP). Stimulation with ephrinA1, a ligand for EphA2, induced further oligomerization and activation of EphA2FL-GFP. The SAM domain deletion mutant, EphA2ΔS-GFP, also underwent further oligomerization upon ephrinA1 stimulation, but the oligomers were larger than those observed for EphA2FL-GFP. Based on these results, we conclude that the EphA2 SAM domain inhibits kinase activity by reducing receptor oligomerization.

Of the 58 transmembrane protein receptor tyrosine kinases (RTKs) in the human genome, 14 are Eph receptors, constituting the largest subfamily of RTKs. They are divided into EphA and EphB subclasses that bind to GPI-anchored ephrin-A and transmembrane ephrin-B ligands, respectively, with some exceptions^1^,^2^,^3^. The Eph/ephrin system mediates cell-cell contact signaling, which takes place in a bidirectional manner through either ephrin-Eph forward signaling or Eph-ephrin reverse signaling^4^. Extensive early studies established the Eph/ephrin system as a versatile and essential regulator of developmental and disease processes^2^,^5^,^6^. In embryonic development, Eph/ephrin interactions regulate cell adhesion and segregation, and also enforce tissue patterning. Dysregulation of the Eph/ephrin system contributes to diverse disease processes including cataracts, neurological disorders, viral infections as well as cancer^3^,^7^,^8^.

Eph receptors are type-I transmembrane proteins. The extracellular domain (ECD) of Eph contains a highly conserved ligand binding domain (LBD), followed by a cysteine rich domain (CRD) and two fibronectin-type III domains (FN I & II). After the transmembrane (TM) domain, the intracellular domain (ICD) of Eph consists of a juxtamembrane segment (JMS), a kinase domain, a sterile alpha motif (SAM domain) and a PDZ binding motif^9^. The activation of Eph is marked by the elevated phosphorylation level of the tyrosine residues in the JMS and kinase domain^10^ and is also accompanied by internalization and degradation of the receptors^11^,^12^. Like other RTKs, activation of Eph starts with ligand binding, which induces receptor oligomerization and then trans-phosphorylation catalyzed by kinases^13^. Upon ligand binding, two tyrosines in the conserved JMS are phosphorylated, which triggers conformational change of the JMS and releases this segment from an inhibitory interaction with kinase domain. These events allow ATP and substrates to access the active site^14^,^15^,^16^,^17^. In addition to the conformational changes in the JMS, the endocytosis and degradation of Eph upon receptor activation is also an important signature for Eph activation. Finally, ligand binding induces spatial rearrangement of the receptors leading to receptor oligomerization, which drives the trans-phosphorylation of the ICD. Oligomerization has thus become another signature of activation, and has been investigated in detail by several structural studies described below.

Structural studies of the extracellular domain (ECD) of EphA2 in complex with ephrinA5 showed clusters with several binding interfaces^18^,^19^. These interfaces include three regions of contact between the LBD of EphA2 and the RBD of ephrinA5, one between the CRD of EphA2 and one between the FN-1 of EphA2, both with a second EphA2 receptor protein. Based on this crystallographic view, a seeding mechanism for Eph-ephrin signaling platform formation was proposed^18^,^19^. Similar EphA2 clustering interfaces were observed in the crystal structure of an EphA2-ephrinA1 complex^20^,^21^. These studies also suggested that the interface at the CRD mediates the formation of signaling competent EphA2/ephrin clusters. In addition to these interfaces, an LBD-FN2 interface was also observed, suggesting that the EphA2/ephrin cluster recruits inactive EphA2 to form a multi-function signaling platform^21^. Micron scale EphA2/ephrinA1 clusters were also observed by Salaita *et al*. in a reconstituted intermembrane signaling system. EphA2 was expressed by live human breast cancer cells and interacted with laterally mobile ephrinA1 on a underlying supported lipid bilayer^22^. The authors also reported that the malignancy of the cancer cells was correlated to the clustering propensities of EphA2.

On the intracellular side, one feature that distinguishes Eph receptors from all other members of the RTK superfamily is the presence of a sterile alpha motif (or SAM) domain at the C-terminal end. SAM domains are 60 to 90 amino acid structural modules, consisting primarily of alpha helices and are known to mediate homophilic and heterophilic protein interactions^23^,^24^. One well-characterized interaction is between EphA2 and SHIP2 SAM domains that regulates EphA2 endocytosis and cell migration^25^,^26^,^27^. Remarkably, the EphA2 interface is highly positively charged, which leads to considerable configurational degeneracy in protein-protein interactions^28^,^29^. Singh *et al*. used a quantitative FRET method to study the dimerization of EphA3 in osmotically swelled cells^30^. Deletion of the SAM domain from EphA3 weakens the dimerization propensity, indicating that the SAM domain promotes unliganded EphA3 dimerization^30^. However, recently, the same group reported that deletion of the SAM domain has the opposite effect on EphA2. Using quantitative FRET in swollen HEK 293 cells they report that the dimerization propensity of EphA2 increases upon SAM deletion^31^. They also found that deletion of the SAM domain led to elevated autophosphorylation of EphA2^31^. Here we investigate the role of the EphA2 SAM domain in several cancer model cell lines with and without ligand stimulation. We found elevated autophosphorylation of tyrosines in the JMS domain resulting from SAM domain deletion from full length EphA2 receptors. This suggests that the deletion of SAM domain leads to a constitutive activation of EphA2 kinase, which is also supported by cell function assays.

The spatial arrangement of Eph receptors in the cell membrane is an important aspect of regulating the Eph signaling pathway^22^,^32^. Using a fluorescence fluctuation method (fluorescence correlation spectroscopy, FCS), we found that truncation of the EphA2 receptor by the SAM domain leads to increased oligomerization compared to the full length receptor, and causes constitutive activation of EphA2. Ligand stimulation of full length EphA2 (EphA2FL-GFP) also increases the oligomerization of these receptors on the cell surface while ligand stimulation of the SAM domain truncated construct (EphA2ΔS-GFP) leads to oligomers with even larger size. The results illustrate an essential role of the SAM domain in controlling lateral assembly of EphA2 receptors and maintaining the unligated receptors in an inactive/autoinhibited state.

## Results and Discussion

### SAM domain deletion leads to constitutive activation of EphA2

**Figure 1:**
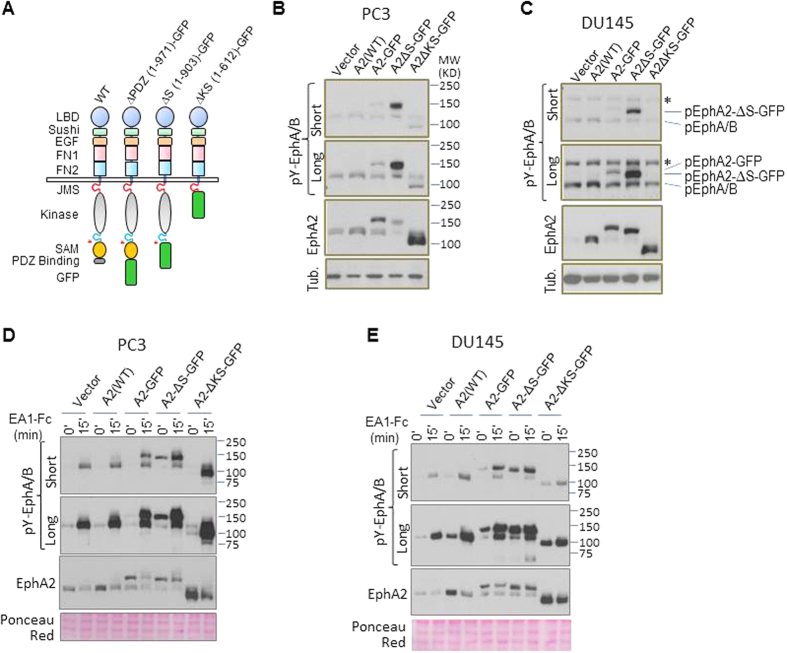
Deletion of the SAM domain led to constitutive activation of EphA2 receptor tyrosine kinase activities. (**A**) Schematic illustration of EpA2 truncation constructs. The constructs were cloned into the LZRS IRES retroviral vector. (**B**) Total cell lysates from PC3 cells expressing the indicated EphA2 truncation mutants were immunoblotted with the indicated antibodies. The pY-EphA/B antibody was raised against the phosphorylated di-tyrosine motif in the juxtamembrane motif conserved in both EphA and EphB receptors. Total EphA2 levels were detected with an antibody against the ectodomain of EphA2. (**C**) Immunoblot of total cell lysate from DU145 cells expressing different truncation constructs. (**D**,**E**) Immunoblot of PC3 (**D**) and DU145 (**E**) cell lysates following stimulation with 3 μg/ml ephrinA1-Fc for 15 min. Ponceau Red staining was used as loading control. Except for the loading controls, full gels containing all bands are included; long and short exposures are also included to show the differences in EphA2 phosphorylation levels.

To interrogate the function of EphA2 SAM domain, we generated three serially truncated mutants that were tagged with enhanced green fluorescence protein (eGFP) in a retroviral expression vector (Fig. 1A). The delta-PDZ mutant contains the full EphA2 sequence except for the last five amino acids for PDZ domain binding (AA 1–971) and was designated as EphA2FL-GFP. The SAM and kinase truncations include up to AA 903 and 612 and were designated as EphA2ΔS-GFP and EphA2ΔKS-GFP respectively. PC3 and DU145 prostate cancer cells were stably transduced with retroviral vectors and subjected to immunoblot analysis for expression and activation status of EphA2 (Fig. 1B–E). In PC3 cells, WT, EphA2FL-GFP and EphA2ΔS-GFP were expressed at equivalent or slightly above the endogenous EphA2 level; EphA2ΔKS-GFP was expressed significantly higher. An antibody against the phospho-dityrosine motif conserved in most Eph receptors (pY-EphA/B) was used to detect activated Eph receptors. Strong constitutive autophosphorylation of EphA2 was detected upon deletion of the SAM domain (Fig. 1B). Wild type or GFP-tagged full length EphA2 showed only low basal activation. The same results were observed in DU145 cells (Fig. 1C). This suggests that the kinase undergoes constitutive activation upon deleting the SAM domain from the receptor sequence.

Stimulation with recombinant ephrinA1-Fc led to strong activation and degradation of endogenous EphA2 in PC3 cells (Fig. 1D), consistent with previous observations^10^. The exogenous WT and EphA2FL-GFP were similarly activated. On top of the high constitutive activation, ligand stimulation caused even further activation of EphA2ΔS-GFP. Intriguingly, despite the lack of the kinase domain, EphA2ΔKS-GFP also showed a high level of ligand-induced phosphorylation, consistent with the location of the phosphorylation sites in the JM domain but also suggests that the phosphorylation may be mediated by co-residing Eph kinases, which have been reported in a previous study^33^. Further work is needed to determine whether other kinase(s) in addition to the endogenous EphA2 may be responsible.

**Figure 2:**
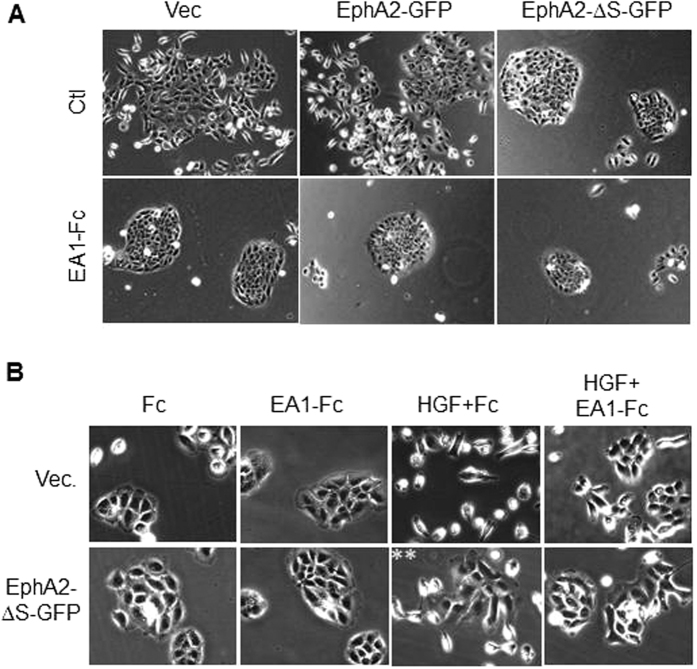
(**A**) Constitutively active EphA2 after SAM deletion caused compaction of DU145 epithelial colonies. DU145 cells were seeded at low density (1,000 cells/well) on 6-well cluster plates and culture for 10 days to allow the emergence of cellular colonies. (**B**) The constitutively active EphA2-ΔS-GFP rendered DU145 cells partially resistant to the HGF-induced cell scattering. Note the Bright field images of DU145 cells with control vector (top row) and EphA2ΔS-GFP (bottom row) treated with Fc (first column), EA1-Fc (second column), HGF + Fc (third column) and HGF + EA1-Fc (forth column). HGF-induced DU145 cell scattering was reduced in vector control cells by EA1-Fc. EphA2-ΔS-GFP expressing cells became more resistant to the HGF-induced scattering as some of the cells still remained attach to each other in the colonies (**).

Next, we examined the functional significance of the constitutively active EphA2. We chose to use DU145 cell line for these studies because of its relatively low level of endogenous EphA2 expression (Fig. 1C), and its epithelial morphology (Supplementary Fig. S1). We had previously shown that ligand (ephrinA1) induced activation of EphA2 on MDCK cells induced the compaction of epithelial cell colonies^34^. DU145 cells that normally form loosely assembled epithelial clusters *in vitro*, also became highly compact upon stimulation with the ephrinA1 (Fig. 2, vector control). Interestingly, expression of SAM deletion mutant EphA2 by itself promoted the compaction of DU145 cells, in keeping with its constitutive activation. The full length EphA2-GFP did not induce the same morphological changes, although it did respond to ephrinA1-Fc stimulation to induce tightly packed colonies. Moreover, DU145 cells expressing EphA2ΔS-GFP conferred partial resistance to HGF-induced cell scattering, a function for the catalytically activated EphA2 previously shown in MDCK cells (Fig. 2B). The constitutive activation of EphA2 upon SAM domain deletion suggests to us that the EphA2 SAM domain may play an inhibitory role in regulating the activation of EphA2. Since the spatial organization of Eph is an important aspect of its activation, our next aim was to investigate the effect of the SAM domain on the lateral organization of Eph in live cancer cell membranes with a time-resolved fluorescence approach.

### Measuring EphA2 receptors in live cells with FCS

**Figure 3:**
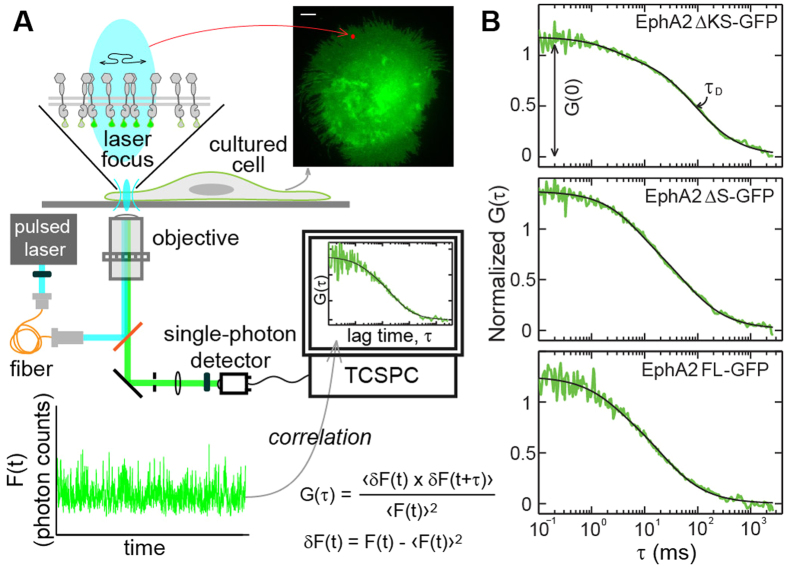
(**A**) Schematic diagram of the FCS experiment. 488 nm pulsed laser beam was focused onto the peripheral membrane of cultured cell to excite the diffusive receptors. The emitted photons were collected by the same objective and directed to a single-photon detector so that the fluorescence fluctuation caused by receptor diffusion can be recorded. Auto-correlation of the fluorescence intensity trace was performed to obtain the auto-correlation function curve (AFC). Insert: epi-fluorescence image of DU145 cell expressing EphA2FL-GFP; the red dot represents the position of the laser beam which is always placed on a flat membrane area. Scale bar is 5 μm. (**B**) Representative AFCs of FCS measurements on DU145 cells with expression of truncation mutant of EphA2 constructs. τ_D_ reports on mobility of the diffusive receptors and is used to calculate the diffusion coefficients. G(0) reports on concentration of the diffusive receptors and is used to calculate molecular brightness.

As with other RTKs, ligand-induced oligomerization of Eph receptors is requisite for catalytic activation^35^,^36^. To understand whether the SAM domain may impact the oligomerization and activation status of EphA2, we turned to fluorescence fluctuation methods to resolve the assembly and oligomerization of EphA2. FCS data was collected from live cells placed in an incubator on the stage of the fluorescence microscope (Fig. 3A). Single cell measurements were made by focusing the excitation laser at lamellipodial regions of the cells (Supplementary Figs S1,S5 and S9) and collecting fluorescence for several 15 second intervals. The resultant fluorescence signal was analyzed to produce FCS data as described in the methods section. Representative FCS curves from different EphA2 truncation constructs are shown in Fig. 2B and in Supplementary Figures S2,S6 and S10.

By measuring the diffusion of the three truncated receptor constructs (Fig. 1A) in DU145 cells with fluorescence correlation spectroscopy (FCS), three kinds of useful information were obtained: molecular brightness, mobility and receptor density. Molecular brightness reflects the average number of photons emitted by each receptor and receptor complex per unit time as it diffuses through the laser focus. In the simplest case, oligomers are brighter than monomers proportional to their sizes, for example, dimers have twice the molecular brightness of monomers, and trimers thrice the amount. However, when an equilibrium between different oligomer states exists, as happens with the receptor proteins in live cell membrane, this relationship will be altered. For example, in a monomer/dimer equilibrium, the apparent molecular brightness will be in between those of monomer and dimer^37^. This makes deciding the size of the oligomers based on molecular brightness data very challenging. Nevertheless, an increase of molecular brightness can still be viewed as a qualitative sign of receptors forming larger assemblies. Receptor mobility, indicated by the diffusion coefficient, is also dependent on the size of the receptor oligomers. Although the absolute scaling of mobility with the size of the diffusing entity is challenging, a decrease in mobility reflects an increase of molecular size due to receptor oligomerization. Large oligomers will diffuse more slowly than small oligomers in the same membrane environment. We measured the mobility of EphA2 by calculating the diffusion coefficient from the decay time of the single cell FCS measurements. FCS also has the ability to measure the average density of diffusing entities^38^, which is challenging to obtain by methods based on fluorescence intensity. By calibrating the diffraction-limited detection area, the two-dimensional receptor density can be obtained under the assumption that the membrane is flat and orthogonal to the optical axis. In this way, FCS allows us to map the receptor density profiles according to oligomerization and activation state with higher precision than classical methods like immunoblots. Molecular brightness and diffusion coefficient data were also plotted against receptor density (Supplementary Figs S4,S8 and S12). Based on these plots, we find that the results do not depend on density over the ranges accessed here (below 300 molecules/μm^2^).

**Figure 4:**
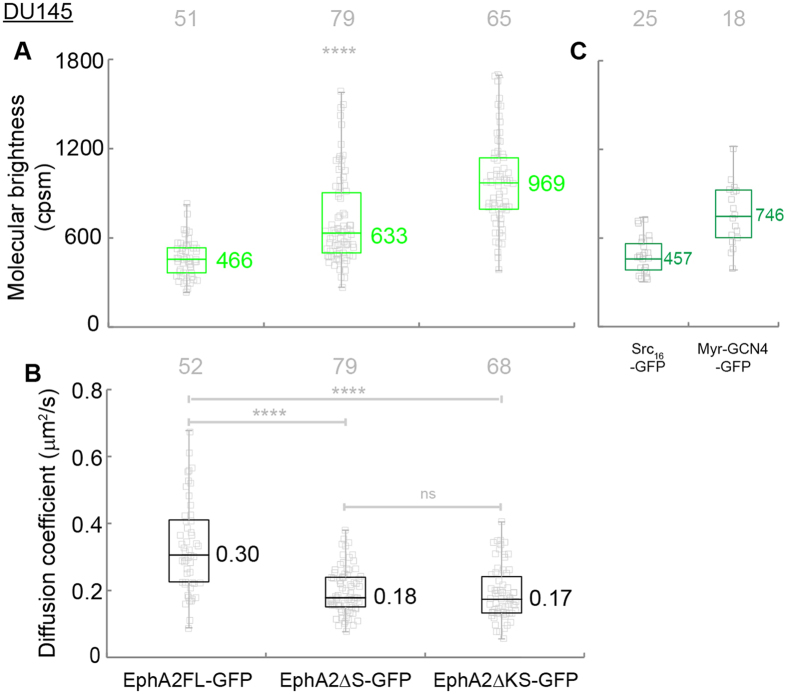
Molecular brightness (**A**, green) and diffusion coefficients (**B**, black) of truncation mutant constructs of EphA2 in DU145 cells. And molecular brightness (**C**, dark green) of Src16-GFP (monomer control) and Myr-GCN4-GFP (dimer control). The median values were reported next to the box plots. Each data point was the average of five 15 s FCS measurements performed on one cell. The grey numbers on top the plots are the total number of cells used. The one-way ANOVA test was performed to obtain the p values (****p < 0.0001, ns: p = 0.9098). The molecular brightness of EphA2ΔS-GFP and EphA2ΔKS-GFP are larger than that of EphA2FL-GFP. EphA2FL-GFP also has the larger diffusion coefficient compared to EphA2ΔKS-GFP and EphA2ΔS-GFP in DU145 cell lines. The results suggest that EphA2ΔS-GFP and EphA2ΔKS-GFP underwent oligomerization beyond that of EphA2FL-GFP in DU145 cell lines.

We first report the molecular brightness (η) values obtained from the single cell FCS data (Fig. 4A). We compared them to two other membrane protein systems measured in live cells on the same instrument and under the same illumination conditions. The first protein is GFP fused to the c-Src membrane localization sequence (Src16-GFP), and has been used in previous studies as a monomer control^39^. The next protein is GFP with GCN4 fused to the cSrc localization sequence (Myr-GCN4-GFP), which has been used as a dimer control. The median value of the single cell molecular brightness data of EphA2FL-GFP is 466 cpsm (Fig. 4A, first column), which is in between the monomer controls (Src16-GFP, 457 cpsm, Fig. 4C, first column) and the dimer controls (Myr-GCN4-GFP, 746 cpsm, Fig. 4C, second column). The data are consistent with EphA2FL-GFP as a monomer, but making such a conclusion based on molecular brightness is not straightforward. For the reasons we stated above, the data are also consistent with ligand-free EphA2 in a monomer-dimer equilibrium. An intensity-based FRET assay was used to illustrate ligand-free dimerization of EphA2 in osmotically swelled cells^40^. In that study, Singh *et al*. reported that ligand free, inactive EphA2 forms dimers with a *K_D_* of 210 receptors/μm^2^. The FCS experiments reported here were performed on cells with an average receptor density of 123 receptors/μm^2^ (Supplementary Fig. S3). This density falls in the lower range of the experiments reported by Singh *et al*., although the density calibration methods were different. From their reported *K_D_* value, the expected dimer fraction at our expression level is 30%. This leads us to conclude that EphA2FL-GFP is in a monomer-dimer equilibrium, with some bias toward the monomeric state. However, a homo-FRET study of EphA2 in Cos-7 cells where Sabet *et al*. showed that ligand free EphA2 remained monomeric even at a high expression level where autonomous activation was detectable^11^. This discrepancy on the lateral organization of unliganded EphA2 receptor is likely due to the difference in experimental conditions, such as expression level, cell type and investigating methods. A more systematic and quantitative investigation is needed to quantify the dynamic associations of unliganded EphA2 in the cell membrane.

### Inhibitory role of SAM domain in EphA2 oligomerization

Dimerization interfaces in the ecto-domain of EphA2 have been identified by previous structural studies^18^,^19^,^20^,^21^. These interfaces include the leucine-zipper like interface at the cysteine rich domain (CRD) involving P221, L223, L254, V255, I257, and the interface between LBD and FN2 domains. Point mutations within the leucine zipper-like interface were shown to destabilize the receptor dimer^40^. In order to investigate the contribution of cytoplasmic domains to receptor dimerization/oligomerization, we performed FCS measurements on two domain deletion constructs, EphA2ΔS-GFP (SAM deletion) and EphA2ΔKS-GFP (kinase and SAM deletion) in DU145 cells as described above for EphA2FL-GFP.

The median values of the single cell molecular brightness data of EphA2ΔS-GFP and EphA2ΔKS-GFP are 633 and 969 cpsm respectively, which are both larger than that of EphA2FL-GFP (Fig. 4A, second and third columns). This indicates that both deletion mutants undergo oligomerization beyond that of EphA2FL-GFP. This could suggest that EphA2ΔS-GFP and EphA2ΔKS-GFP have larger dimeric fraction compared to EphA2FL-GFP or possibly form higher order oligomers, but as we mentioned above, molecular brightness data alone is unable to rigorously quantify the exact size of oligomer. The difference of the molecular brightness between EphA2ΔS-GFP and EphA2ΔΚS-GFP is likely due to different equilibrium distribution of oligomer states. As shown in Fig. 4B, the diffusion coefficients of EphA2ΔS-GFP and EphA2ΔKS-GFP (0.18 and 0.17 μm^2^/s) are significantly smaller than that of EphA2FL-GFP (0.30 μm^2^/s, Fig. 4B, first column). The decrease in mobility supports the interpretation that EphA2ΔS-GFP and EphA2ΔKS-GFP both undergo oligomerization beyond that of EphA2FL-GFP. Together, our data indicate that both EphA2ΔS-GFP and EphA2ΔKS-GFP form oligomers larger than that of EphA2FL-GFP in the cell membrane of DU145. An oligomeric state of EphA2ΔKS-GFP agrees with the reported EphA2 ecto-domain oligomerization^18^,^20^. The oligomeric state of EphA2ΔS-GFP indicates that the kinase domain does not inhibit oligomerization. Comparison with the EphA2FL-GFP data shows that the presence of the SAM domain prevents oligomerization. It also shows that the constitutive activation of the kinase in the SAM domain deleted construct is likely induced by receptor oligomerization.

### Effect of Endogenous EphA2 receptors on FCS measurements

**Figure 5:**
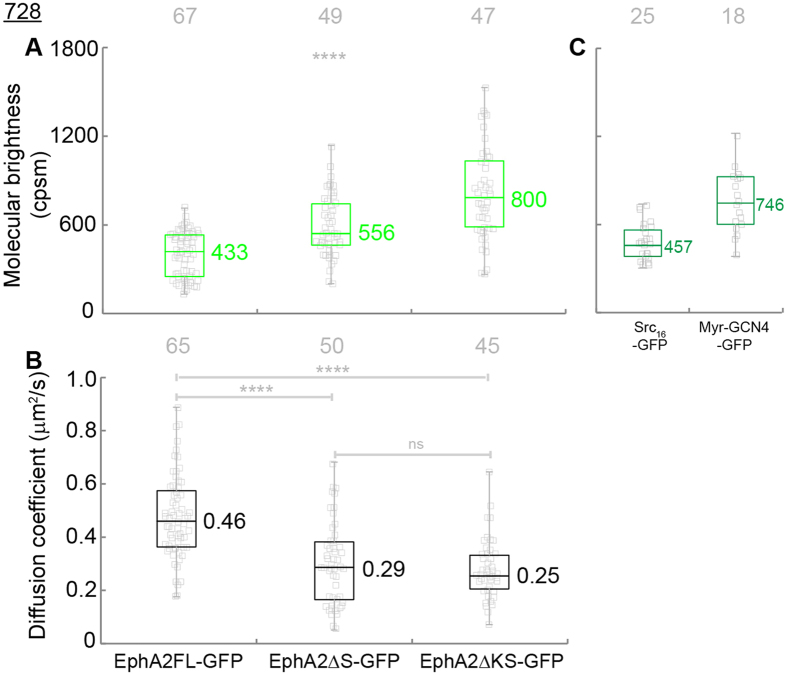
Molecular brightness (**A**, green) and diffusion coefficients (**B**, black) of truncation mutant constructs of EphA2 in 728 cells. And molecular brightness (**C**, dark green) of Src16-GFP (monomer control) and Myr-GCN4-GFP (dimer control). The median values were reported next to the box plots. Each data point was the average of five 15 s FCS measurements performed on one cell. The grey numbers on top the plots are the total number of cells used. The one-way ANOVA test was performed to obtain the p values (****p < 0.0001, ns: p = 0.6643). The molecular brightness of EphA2ΔS-GFP and EphA2ΔKS-GFP are larger than that of EphA2FL-GFP. EphA2FL-GFP also has the larger diffusion coefficient compared to EphA2ΔKS-GFP and EphA2ΔS-GFP in 728 cell lines. The results suggest that EphA2ΔS-GFP and EphA2ΔKS-GFP underwent oligomerization beyond that of EphA2FL-GFP in 728 cell lines.

As a fluorescence-based method, FCS only counts receptors tagged with GFP. However, endogenous receptors with no GFP tags could bias the results and lead to the wrong conclusions. For instance, one dimer consisting of a labeled and an unlabeled protein would have half the molecular brightness of that consisting of two labeled proteins, which could seriously compromise the accuracy of the technique. Despite the fact that DU145 cells have relatively low endogenous EphA2, we sought to investigate the lateral organization of EphA2 constructs without any endogenous EphA2 to verify the unique function of the EphA2 SAM domain. To this end we utilized 728 cells, a mouse squamous cell line derived from an EphA1/EphA2 double knockout mouse (see Methods). The same retroviral vectors used for the DU145 cell experiments were used to stably transduce 728 cells, resulting in EphA1/EphA2 double knockout 728 cells with stable expression of the GFP-tagged EphA2 constructs. The morphology of 728 cells with extended lamellipodia makes them suitable for FCS measurements (Supplementary Fig. S5). FCS measurements were performed on 728 cells and the resulting diffusion coefficients and molecular brightness parameters are summarized in Fig. 5.

The molecular brightness of EphA2FL-GFP in 728 cells is 433 cpsm (Fig. 5A, first column) which is similar to that in DU145 cells (466 cpsm, Fig. 4A). Based on the molecular brightness data we conclude that EphA2FL-GFP is in the same monomer/dimer state in 728 cells as it is in DU145 cells. The molecular brightness of EphA2ΔS-GFP and EphA2ΔKS-GFP are 556 cpsm and 800 cpsm respectively (Fig. 5A, second and third columns), indicating that the receptors undergo increased oligomerization compared to EphA2FL-GFP. The diffusion coefficients of EphA2ΔS-GFP and EphA2ΔKS-GFP in 728 cells are 0.29 and 0.25 μm^2^/s (Fig. 5B, second and third column). Compared to monomeric EphA2FL-GFP (D: 0.46 μm^2^/s), the mobility of EphA2ΔS-GFP and EphA2ΔKS-GFP is decreased, which supports increased oligomerization of these two constructs. These observations lead to the same conclusions as in DU145 cells. The similarity in the results suggests that the expression level of endogenous EphA2 in DU145 cells is much lower than that of the GFP-tagged EphA2 constructs. Together, the FCS experiments in DU145 and 728 cells confirm that deletion of SAM domain leads to oligomerization of EphA2ΔS-GFP and EphA2ΔΚS-GFP beyond that of EphA2FL-GFP.

### Activation of EphA2 with ephrinA1 in 728 cells

EphA2 is activated by its cognate ligands, the ephrins. Structural studies have shown that the ligand binding domain (LBD) of EphA2 and the receptor binding domain (RBD) of ephrin form a circular tetramer consisting of two LBDs and two RBDs involving three interfaces^20^. Together with the additional oligomerization interface in the leucine zipper-like CRD, the RBD of ephrin and the ecto-domain of EphA2 could form higher order oligomers. Based on these structures, a steric “seeding” mechanism was proposed, in which ephrin binding resulted in an Eph-ephrin oligomer as “nucleation point” and then has the ability to trigger more widespread EphA2 recruitment. This structural model is in agreement with live cell imaging results, supporting the conclusion that ephrin binding would trigger the formation of extended assemblies of EphA2 at the conjunction of cells^22^,^32^.

We set out to investigate the lateral organization of ephrinA1-bound EphA2 constructs using fluorescence imaging and fluctuation spectroscopy. Ligand activation was done with ephrinA1-Fc (EA1Fc), a soluble Eph ligand resulting from the fusion of ephrinA1 to the heavy chain of human IgG1. As demonstrated in Fig. 1, soluble EA1-Fc treatment caused down-regulation of EphA2 and elevation of phosphorylation level of tyrosine, which both indicate activation of EphA2 kinase. We treat the 728 cells with 1 μg/ml EA1-Fc for 20 min before beginning the imaging and FCS measurements. Upon activation, the population of EphA2 on the plasma membrane decreased by a factor of 5 (Supplementary figs S7 and S11). The decrease in receptor density is likely due to the internalization of ligand-activated EphA2.

**Figure 6:**
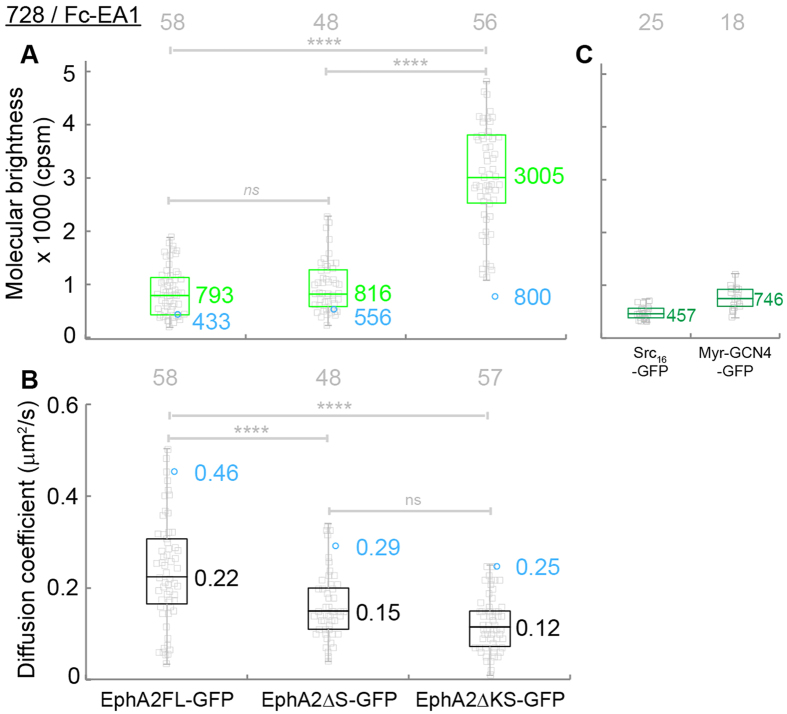
Molecular brightness (**A**, green) and diffusion coefficients (**B**, black) of truncation mutant constructs of EphA2 in 728 cells with EA1-Fc treatment. And molecular brightness (**C**, dark green) of Src16-GFP (monomer control) and Myr-GCN4-GFP (dimer control). The median values were reported next to the box plots. Each data point was the average of five 15 s FCS measurements performed on one cell. The blue circles and values are the diffusion coefficients and molecular brightness of EphA2 constructs before Fc-EA1 treatment. The grey numbers on top the plots are the total number of cells used. The one-way ANOVA test was performed to obtain the p values (****p < 0.0001, *ns*: p = 0.0617, ns: p = 0.6878). The molecular brightness of the three constructs all increased upon Fc-EA1 binding. The diffusion coefficients of the three EphA2 constructs all decreased ~50% indicating the formation of larger assemblies of EphA2 receptors, which agrees with the increase of molecular brightness as larger clusters are brighter particles with slower motion. These observations demonstrated EphA2 constructs undergo further oligomerization upon Fc-EA1 binding.

Upon EA1-Fc binding, the molecular brightness of all three EphA2 constructs increased while the diffusion coefficients decreased (Fig. 6A and B). This observation agrees with expected EphA2 oligomerization upon ligand binding. The molecular brightness of EphA2FL-GFP almost doubled, from 433 cpsm to 793 cpsm (Fig. 6A, first column), while the diffusion coefficient decreased more than 50%, from 0.46 μm^2^/s to 0.22 μm^2^/s (Fig. 6B, first column) suggesting that EphA2FL-GFP form larger oligomers upon ligand binding. Deciding the degree of oligomerization is very difficult based on single-color FCS measurements. Future work will investigate the oligomerization state directly by dual-color FCCS, which helped solve similar problem in the case of EGFR^41^.

In order to provide a relevant comparison with the crystal structure studies, we also performed measurements on EA1-Fc stimulated EphA2ΔS-GFP and EphA2ΔKS-GFP. In the case of EphA2ΔKS-GFP there is no kinase nor SAM domain, which makes it very similar to the ecto-domain construct reported in the structural studies^18^,^19^,^20^,^21^. In the presence of EA1-Fc, the molecular brightness of EphA2ΔS-GFP and EphA2ΔKS-GFP both increased significantly, suggesting that compared to ligand-free oligomers, these constructs undergo further oligomerization. The molecular brightness of EphA2ΔKS-GFP increased from 800 cpsm to 3005 cpsm (Fig. 6A, third column). Based on this dramatic increase in brightness, it is evident that EphA2ΔKS-GFP underwent extensive oligomerization upon EA1-Fc binding and formed larger clusters than its ligand free state (800 cpsm). Meanwhile, upon ligand binding, the diffusion coefficients of EphA2ΔS-GFP and EphA2ΔKS-GFP both decreased ~50% comparing to their ligand-free state (from 0.29 to 0.15 μm^2^/s and from 0.25 and 0.12 μm^2^/s, Fig. 6B, second and third columns). This suggests that EphA2ΔS-GFP and EphA2ΔKS-GFP form larger-sized oligomers upon EA1-Fc binding compared to the ligand-free state. This observation of cluster formation agrees with the prediction of the steric seeding model and the crystal structure data that the model is based on^18^,^19^.

Interestingly, the ligand bound EphA2FL-GFP cluster has higher mobility and lower brightness than those of the clusters of EphA2ΔS-GFP. This means that EA1-Fc binding causes EphA2FL-GFP to oligomerize to a lesser extent than EphA2ΔS-GFP. This observation illustrates that SAM domain, in addition to inhibiting oligomerization of the ligand-free receptor, also has the ability to reduce EphA2 clustering upon ligand binding.

## Conclusions

In order to understand the function of the cytoplasmic domain of EphA2 in receptor activation, we carried out an investigation of EphA2 and two intracellular domain deletion mutants: EphA2FL the full length EphA2 sequence, EphA2ΔS of which SAM domain is deleted from EphA2FL and EphA2ΔKS of which kinase and SAM domain are both deleted. Immunoblots demonstrated that deletion of SAM domain from EphA2 leads to elevated phosphorylation level of tyrosine. Together with the cell function assays, we concluded that the deletion of the SAM domain caused constitutive activation of the EphA2 RTK.

FCS measurements were performed on live cancer cells with stable expression of GFP-tagged EphA2 mutation constructs (DU145), and mouse epithelial tumor cells with EphA1/EphA2 gene knockout and stable expression of GFP-tagged EphA2 mutation constructs (728). Based on the FCS measurements, we found that full length EphA2 is not significantly dimerized in DU145 cells (with endogenous EphA2) and 728 cells (without endogenous EphA2). The EphA2 constructs with deletion of both kinase and SAM domains (EphA2ΔKS) and deletion of SAM domain (EphA2ΔS) underwent increased oligomerization compared to EphA2FL-GFP in both DU145 and 728 cells. These results demonstrated that the presence of the SAM domain reduces ligand-free oligomerization. The FCS results indicate that the constitutive activation of the kinase of the SAM domain deletion construct is induced by receptor oligomerization. These results add mechanistic insight into the activity of EphA2 and the inhibitory role of the SAM domain in EphA2 oligomerization.

We also performed FCS measurements on 728 cells with ephrinA1-Fc (EA1-Fc) treatment. The results indicate that EA1-Fc binding causes EphA2FL-GFP to form oligomers, while the preoligomerized EphA2ΔS-GFP and EphA2ΔKS-GFP form even larger clusters. The presence of the SAM domain in EphA2FL-GFP prevented the receptors from forming clusters as large as the EA1-induced EphA2ΔS-GFP and EphA2ΔKS-GFP clusters. This is consistent with the inhibitory tendency of SAM domain towards EphA2 oligomerization observed with ligand-free receptors.

This work illustrated the unique function of SAM domain in regulating the lateral organization and activity of EphA2. Within the RTK protein family, Eph receptors are the only ones that have a C-terminal SAM domain. Apart from the known function of SAM binding with other signaling proteins that contains SH2, for example SHIP2^26^, and when phosphorylated, also Grb7^27^, our work suggests a novel regulatory function of SAM domain towards Eph RTKs. Although the structural details of this regulatory function are still not clear, we can conclude that the SAM domain creates a unique set of constraints on Eph receptor kinase activity compared to other RTKs lacking the SAM domain^42^. Moreover, the SAM domain has been reported to promote oligomerization and activation of EphA3^30^, which suggests an unexpected diversity of function within the Eph family. All of these make the SAM domain an important factor in decoding the function of Eph receptors.

## Methods

### Establishment of skin tumor cells from EphA1/EphA2 double knockout mice

*EphA1* knockout mice were generated through the insertion of an internal ribosome entry site (IRES)-human placental alkaline phosphatase (ALPP) reporter cassette into exon II of the *EphA1* gene as described previously^43^. They were obtained from Dr. Andrew Boyd. The CN3 line of *EphA2* mutant mice were generated by insertion of a gene trap vector into intron 1 of the EphA2 gene, which eliminated all EphA2 gene expression^44^. They were crossed with each other to generate EphA1^−/−^:EphA2^−/−^ double knockout mice. The mice were subject to DMBA/TPA two stage carcinogenesis as described previously^45^. To establish skin cell lines, skin tumors were cut into less than 1 mm pieces at room temperature, and were digested with 3.5 mg/ml collagenase in Hanks Balanced Saline Solution (HBSS, Ca++, Mg++, Gibco) 1 hour at 37 °C on rocking plate. After centrifugation at 400 g for 10 min at 4 °C, the pellets were washed in 2% FBS in PBS and passed through a 70-micron filter. Tumor cells were plated on wells with γ-irradiated 3T3 feeder cells in keratinocyte medium (MEM medium supplemented with 10% FBS, 0.4 μg/ml hydrocortisone, 5 μg/ml insulin, 10 ng m/l EGF, 2 × 10^−9^ M T3, 1% penicillin/streptomycin, 2 mM l-glutamine). After one round passage, tumor cells were maintained in keratinocyte medium on plates precoated with 25 μg/mL collagen I.

All procedures involving mice were performed in accordance with guidelines set forth by the American Association for Accreditation of Laboratory Animal Care and the USPHS “Policy on Humane Care and Use of Laboratory Animals”. Studies were approved and supervised by The Case Western Reserve University Institutional Animal Care and Use Committee.

### Retrovirus-mediated gene transduction

Human EphA2 cDNA was obtained from Dr. Tony Hunter. Full length (FL, AA 1–971), SAM domain deletion (ΔS, 1–903), or cytoplasmic deletion including the kinase and SAM domain (ΔKS, 1–612) were amplified with PCR with appropriate primers and cloned into pEGFP-C1 plasmid in frame with the eGFP coding sequence. The entire eGFP fusion fragments were then inserted into LZRS-Pac retrovirus vector. They were then transfected into Phoenix retroviral packaging cells to produce the retrovirus. DU145, PC3 and 728 cells were infected with retroviral-mediated gene transfer in the presence of 6 µg/ml polybrene and selected in the presence of 1 mg/ml puromycin.

### Ligand stimulation, immunoprecipitation, immunoblotting and antibodies

Subconfluent DU145, PC3 and 728 cells were treated with 3 μg/ml ephrinA1-Fc^46^. At indicated times, cells were lysed for 30 min at 4 °C in modified RIPA buffer (20 mM Tris, pH 7.4, 120 mM NaCl, 1% Triton X-100, 0.5% sodium deoxycholate, 0.1% SDS, 10% glycerol, 5 mM EDTA, 50 mM NaF, 0.5 mM Na3VO4, and protease inhibitors, including 1 mM phenylmethylsulphonyl fluoride, and 2 mg/ml each of aprotinin and leupeptin). Lysates were clarified at 13,000 g for 5 min, and either analyzed immediately or stored at −80 °C. Immunoprecipitations and immunoblot were carried out essentially as described^46^. Antibodies used include goat anti-EphA2 ectodomain antibody (R&D, AF3035) and mouse anti-tubulin (Sigma Aldrich, T5168). Rabbit anti-phospho-EphA/B antibody was raised against the phosphorylated di-tyrosine motif in the conserved juxtamembrane motif of Eph receptors as described previously^47^.

### Cell scattering assay

Cell scattering assays were carried out as described^34^. Briefly DU145 cells were seeded at low density and were cultured for one week to allow the formation of individual colonies. Hepatocyte growth factor (HGF), also know scatter factor, was added at 10 ng/ml to induce epithelial cell scattering in the presence or absence of ephrin-A1-Fc at 3 μg/ml. Phase contrast images were taken after overnight culture.

### Fluorescence Instrumentation

Fluorescence imaging and FCS measurements were performed on a customized Nikon Eclipse Ti inverted microscope (Nikon Corp., Tokyo, Japan) with home-built pulsed interleaved excitation and time-correlated single-photon detection (Fig. 3A). A continuum white light laser (9.7 MHz, SuperK NKT Photonics, Birkerod, Denmark) is used as excitation laser source. The source has an internal pulse picker that allows us to set the pulse duration to 5 ps. A wavelength splitter inside the emission box picks off a 488 nm ± 10 nm beam and the rest of the white light is directed to a beam dump. The 488 nm beam pass through narrow-band excitation filters (488: LL01-488-12.5; Semrock, Rochester, NY) before being coupled into single-mode optical fibers (488: QPMJ-3AF3U-488-3.5/125-3AS-18-1-SP; OZ Optics, Ottawa, Ontario). The beam exits the fiber and is collimated with infinity corrected objective lenses (L10x, Newport, Irvine, CA). Continuously variable ND filter is placed after the lens so that selected laser power can be set independently for the beam. The laser beams is sent into the optical path of the microscope. A customized TIRF filter cube (91032, Chroma Technology Corp., Bellows Falls, VT) with a two-color dichroic mirror and laser blocking filer (zt488/561rpc and zet488/561 m, Chroma Technology Corp., Bellows Falls, VT) is used to allow the beam being fed to the objective. A 100X TIRF (oil) objective, NA 1.49, (Nikon Corp., Tokyo, Japan) was used to focus the excitation beam on the cell sample and collect the emitted photons. On-stage incubator is used to keep the cell sample under 37 °C during the measurements. For time-correlated single photon detection, the emitted photons pass through a 50 μm pinhole placed at the output port of the microscope to achieve confocal detection. The beam is collimated with a 100 mm focal length achromatic lens (AC254-100-A-ML, Thorlabs Inc., Newton, NJ). The beam is directed through a 520/44 nm emission filter (FF01-520/44-25, Semrock, Rochester, NY) to obtain a green (520 nm) emission beam. The emission beam is focused to a single photon avalanche diode (SPAD) detector (Micro Photon Devices, Bolzano, Italy) with a time-resolution of 30 ps and 50 μm^2^ active area, 25 dark counts per second. Signals collected by the detector are recorded with a four-channelrouted time-correlated single photon counting (TCSPC) card (Picoharp 300, PicoQuant, Berlin, Germany) which is synchronized with the white light laser source. Data recorded by TCSPC card is input to a computer for correlation with a home-written Matlab script.

### FCS measurements and data analysis

Cells were cultured in 10% FBS/DMEM on collagen coated plates. For activation, cells were incubated in 1 μg/mL EA1-Fc/Opti-MEM for 20 min prior to FCS experiment. FCS measurements were performed on live cells at 37 °C. The 488 nm excitation laser was set at a power of 300 nW and only focus at flat membrane area for data collections. Each data point was the average of five 15 s measurements at the same spot. Results from 50~90 cells (data points) were shown in the box plot figure. The recorded fluorescence fluctuation signals (*F(t*)) are auto-correlated with a function as


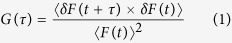



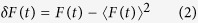


where *τ* is the lag time, *G(τ*) is the auto-correlation function and 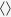 stands for time average. The

resulted correlation function curve was fitted with


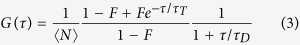


where *N* is average number of fluorescent particles, *τ*_*D*_ is the average dwell time of fluorescent

particles within the detection volume, F is the fraction of molecules in the triplet state, *τ*_*T*_ is the triplet

relaxation time. The molecular brightness (η) and diffusion coefficient (*D*) was calculated based on *N* and

*τ*_*D*_, following the equations


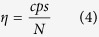



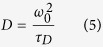


where *cps* is the photon counts recorded per second and ω_0_ is the waist of the laser focus.

## Additional Information

**How to cite this article**: Shi, X. *et al*. A role of the SAM domain in EphA2 receptor activation. *Sci. Rep.* **7**, 45084; doi: 10.1038/srep45084 (2017).

**Publisher's note:** Springer Nature remains neutral with regard to jurisdictional claims in published maps and institutional affiliations.

## Supplementary Material

Supplementary Information
