# Supplementary material for: A role of the SAM domain in EphA2 receptor activation
Source: Sci Rep. 2017 Mar 24;7:45084. doi: 10.1038/srep45084 (PMC5364462; doi:10.1038/srep45084)
Supplement: Supplementary Information [file srep45084-s1.pdf]

Supplementary Information for:

A role of the SAM domain in EphA2 receptor activation

Xiaojun Shi [§], Vera Hapiak [‡,|,¶], Ji Zheng [‡,|,¶], Jeanine Muller-Greven [‡], Deanna Bowman [§], Ryan Lingerak [◊], Matthias Buck [‡,|,*], Bing-Cheng Wang [‡,|,¶,†], and Adam W. Smith [§,†]

Departments of [§]Chemistry and [◊]Biology, University of Akron, Akron OH 44325

Departments of [‡]Physiology and Biophysics, [|]Pharmacology, and [*]Neurosciences, Case Western Reserve University, Cleveland, Ohio 44106

[¶]Rammelkamp Center for Research, MetroHealth Medical Center, Cleveland, Ohio 44109

[†]To whom correspondence should be addressed: asmith5@uakron.edu and bxw14@case.edu

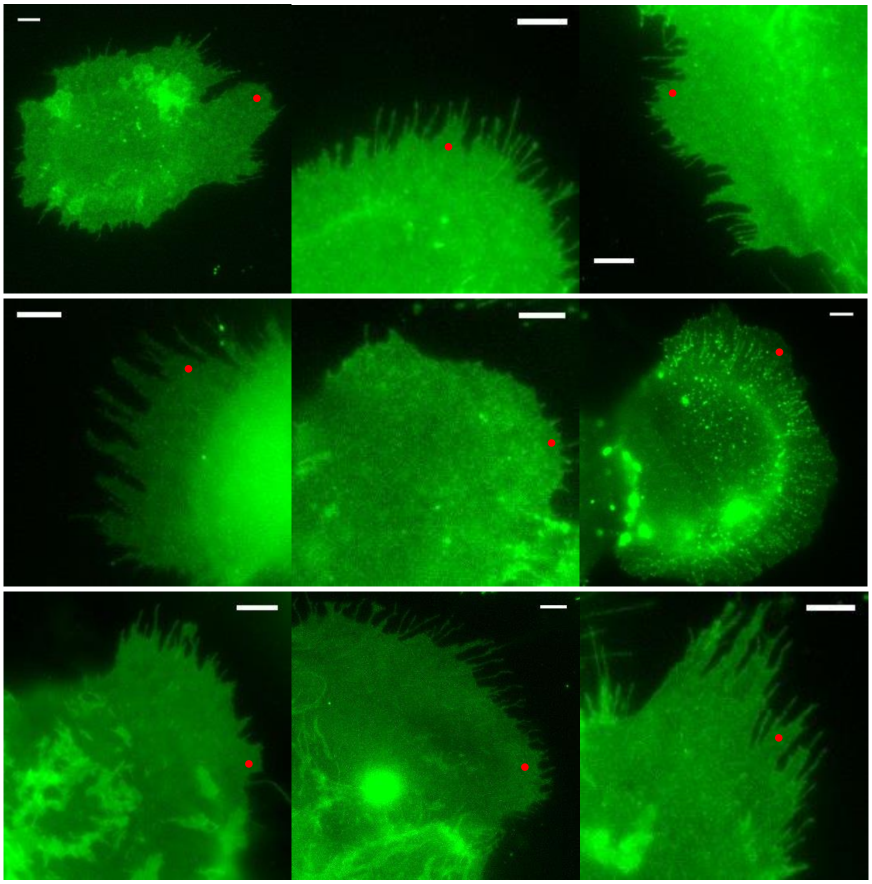

Figure S1. Epi-fluorescence images of DU145 cells with EphA2FL-GFP (upper row), EphA2ΔS-GFP (middle row) and EphA2ΔKS-GFP (lower row) for FCS measurements. The positions of laser beam were marked as red dots. Laser beam was always focused at membrane area on the edge of the cell, avoiding any bright features. All scale bars are 5 μm.

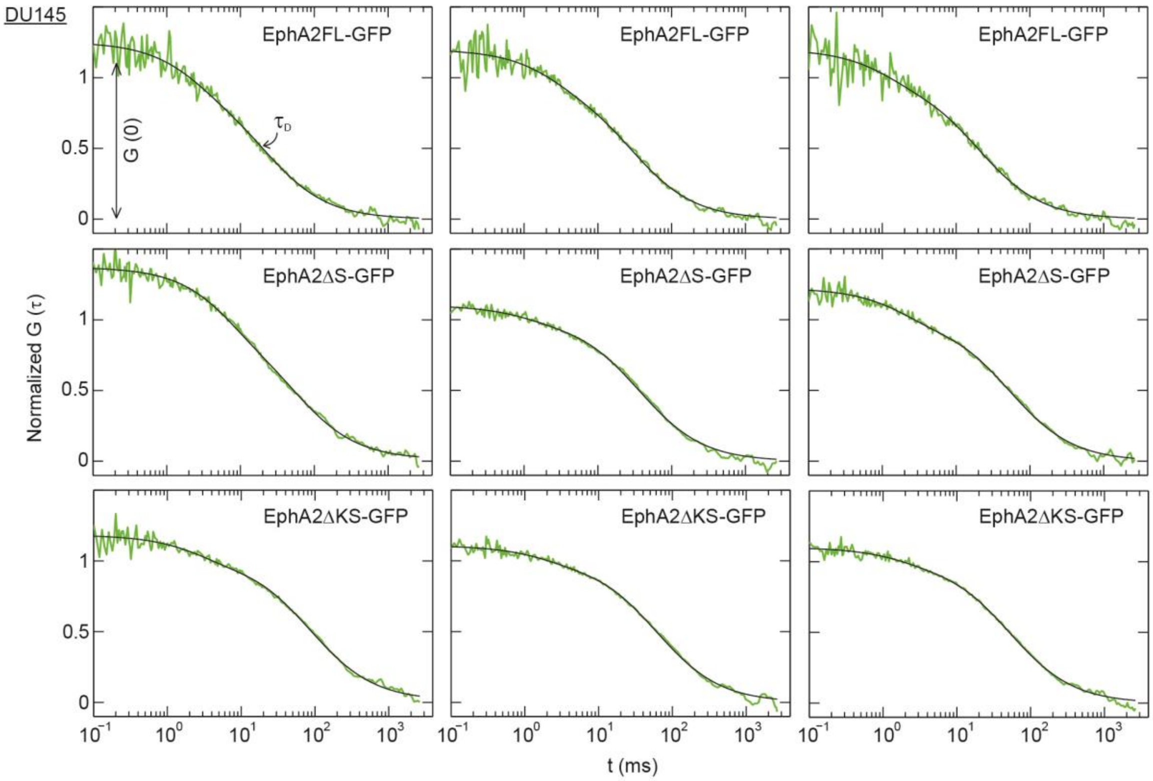

Figure S2. Representative auto-correlation curves of FCS measurements on DU145 cells with expression of truncation mutant of EphA2 constructs. τ_D reports on mobility of the diffusive receptors and is used to calculate the diffusion coefficients. G (0) reports on concentration of the diffusive receptors and is used to calculate molecular brightness.

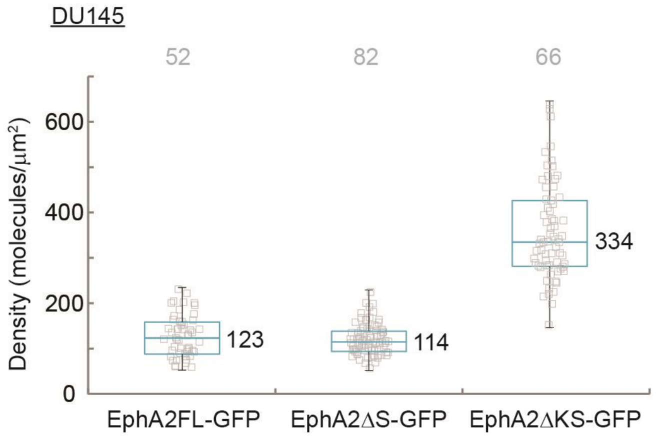

Figure S3. Density of truncation mutant constructs of EphA2 in DU145 cells. The median values were reported next to the box plots. Each data point was the average of five 15s FCS measurements performed on one cell. The grey numbers on top the plots are the total number of cells used.

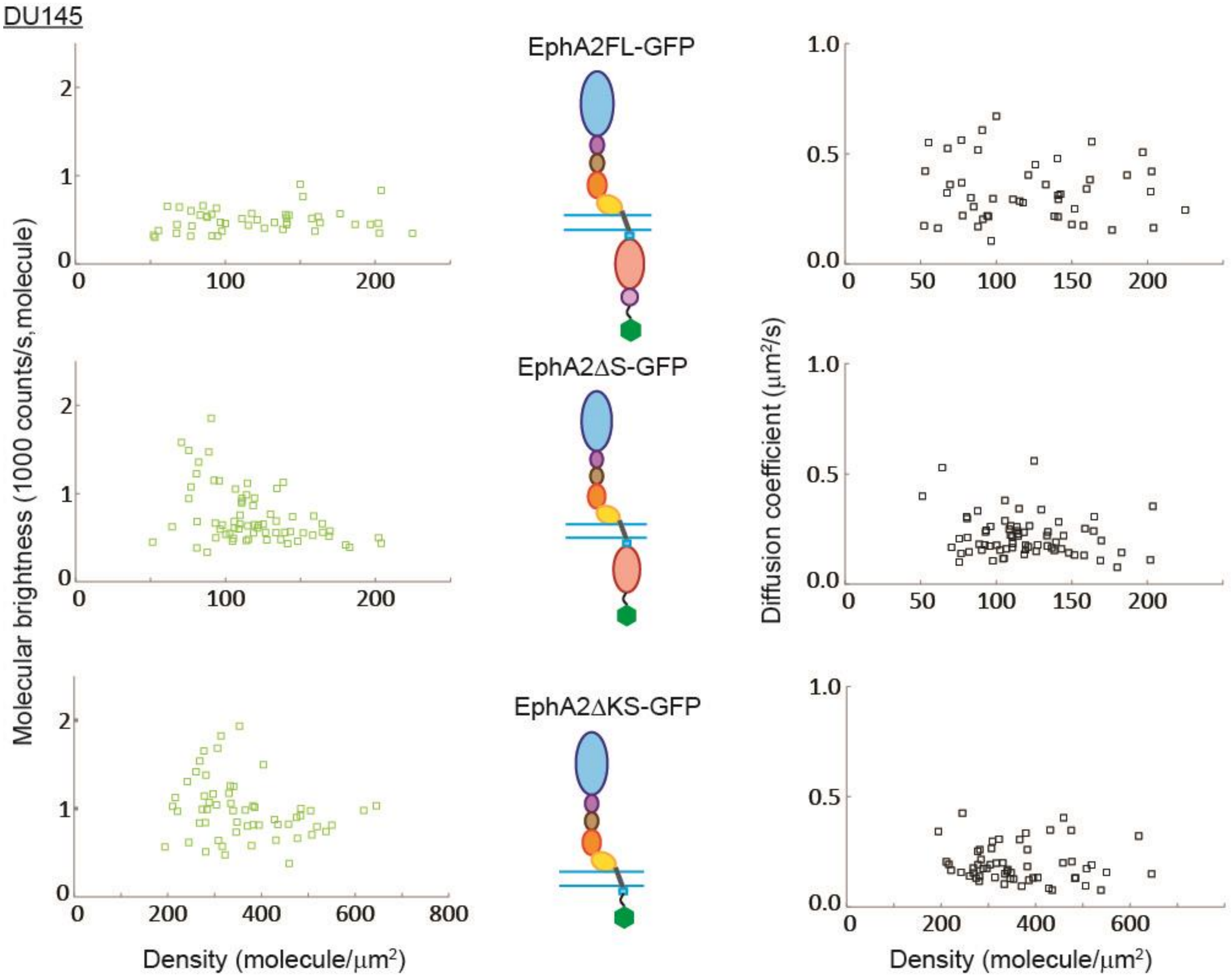

Figure S4. Molecular brightness (left, green) and diffusion coefficient (right, black) of truncation mutant constructs of EphA2 in DU145 cells against receptor density. Each data point was the average of five 15s FCS measurements performed on one cell. There is no obvious dependence of molecular brightness or diffusion coefficient on receptor density.

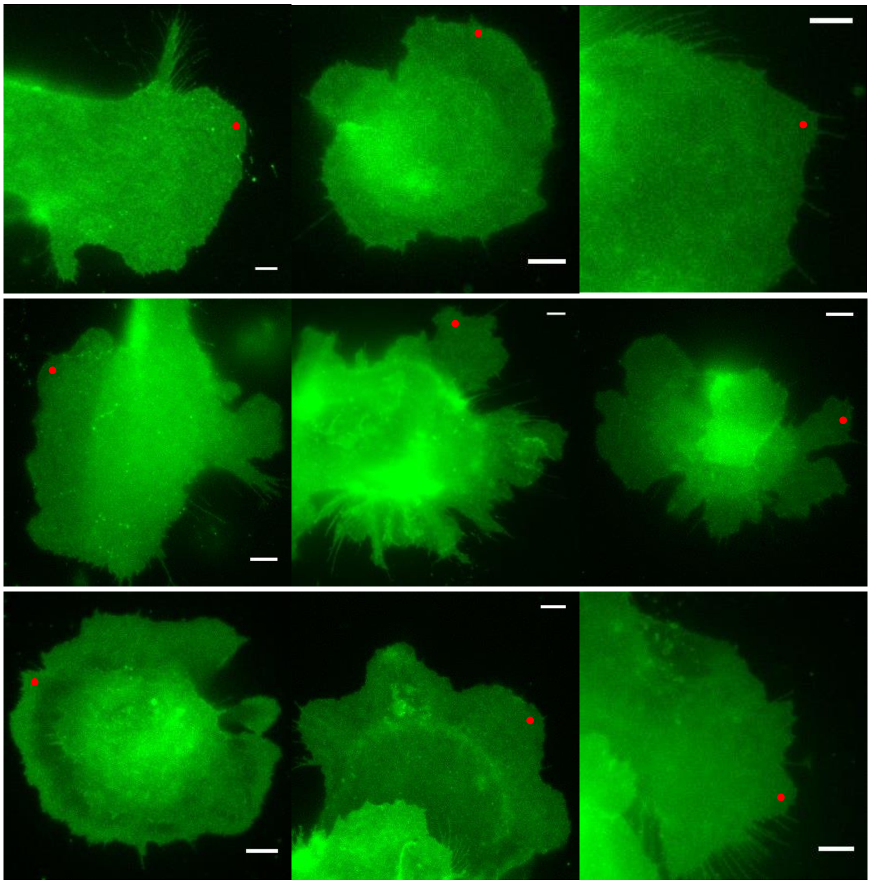

Figure S5. Epi-fluorescence images of 728 cells with EphA2FL-GFP (upper row), EphA2ΔS-GFP (middle row) and EphA2ΔKS-GFP (lower row) for FCS measurements. The positions of laser beam were marked as red dots. Laser beam was always focused at membrane area on the edge of the cell, avoiding any bright features. All scale bars are 5 μm.

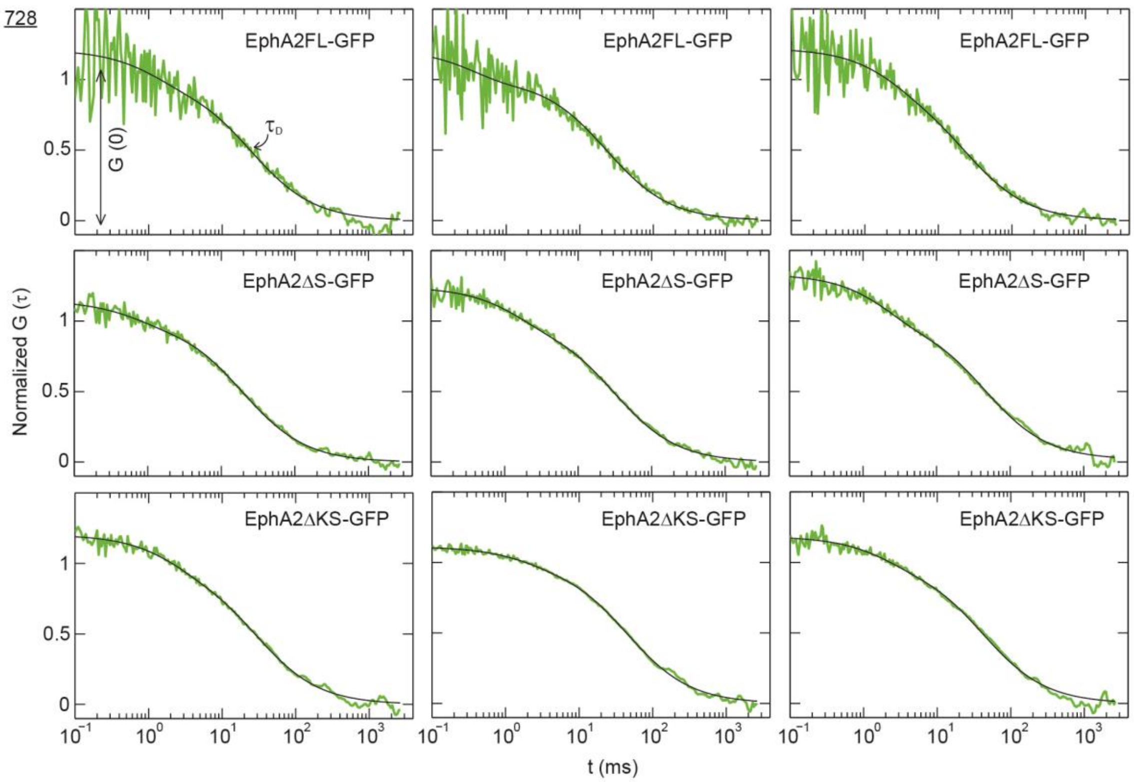

Figure S6. Representative auto-correlation curves of FCS measurements on 728 cells with expression of truncation mutant of EphA2 constructs. τ_D reports on mobility of the diffusive receptors and is used to calculate the diffusion coefficients. G (0) reports on concentration of the diffusive receptors and is used to calculate molecular brightness.

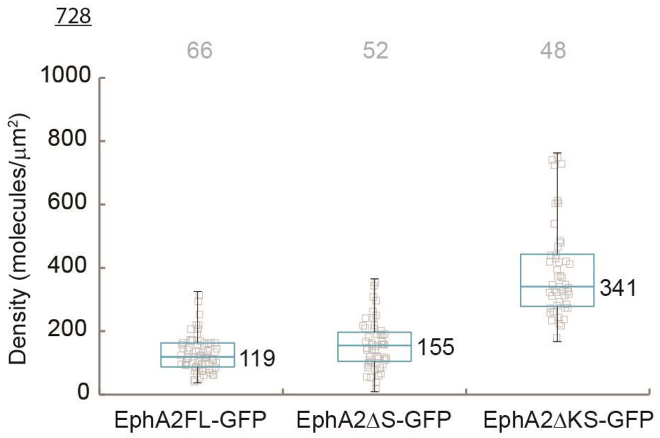

Figure S7. Density of truncation mutant constructs of EphA2 in 728 cells. The median values were reported next to the box plots. Each data point was the average of five 15s FCS measurements performed on one cell. The grey numbers on top the plots are the total number of cells used.

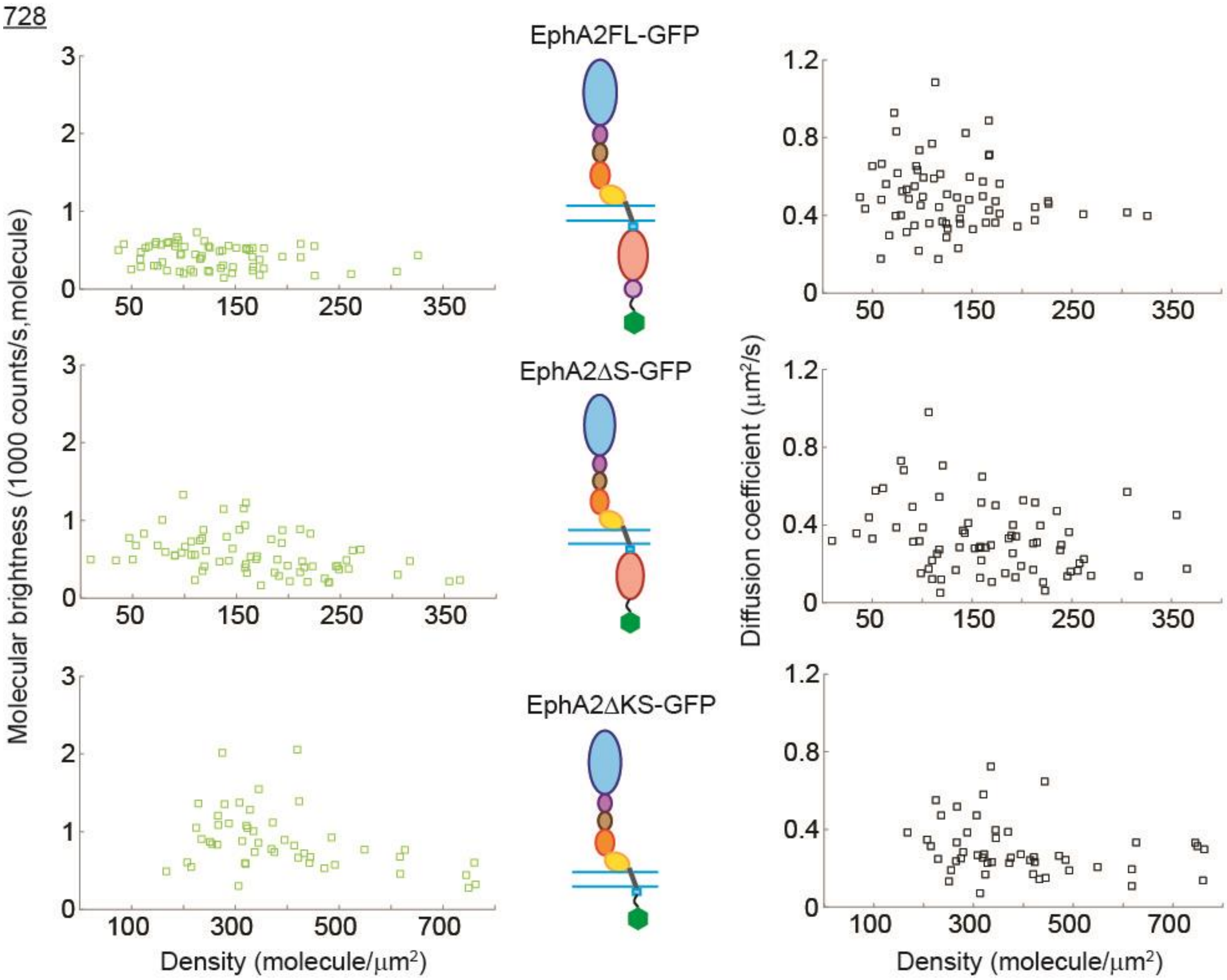

Figure S8. Molecular brightness (left, green) and diffusion coefficient (right, black) of truncation mutant constructs of EphA2 in 728 cells against receptor density. Each data point was the average of five 15s FCS measurements performed on one cell. There is no obvious dependence of molecular brightness or diffusion coefficient on receptor density.

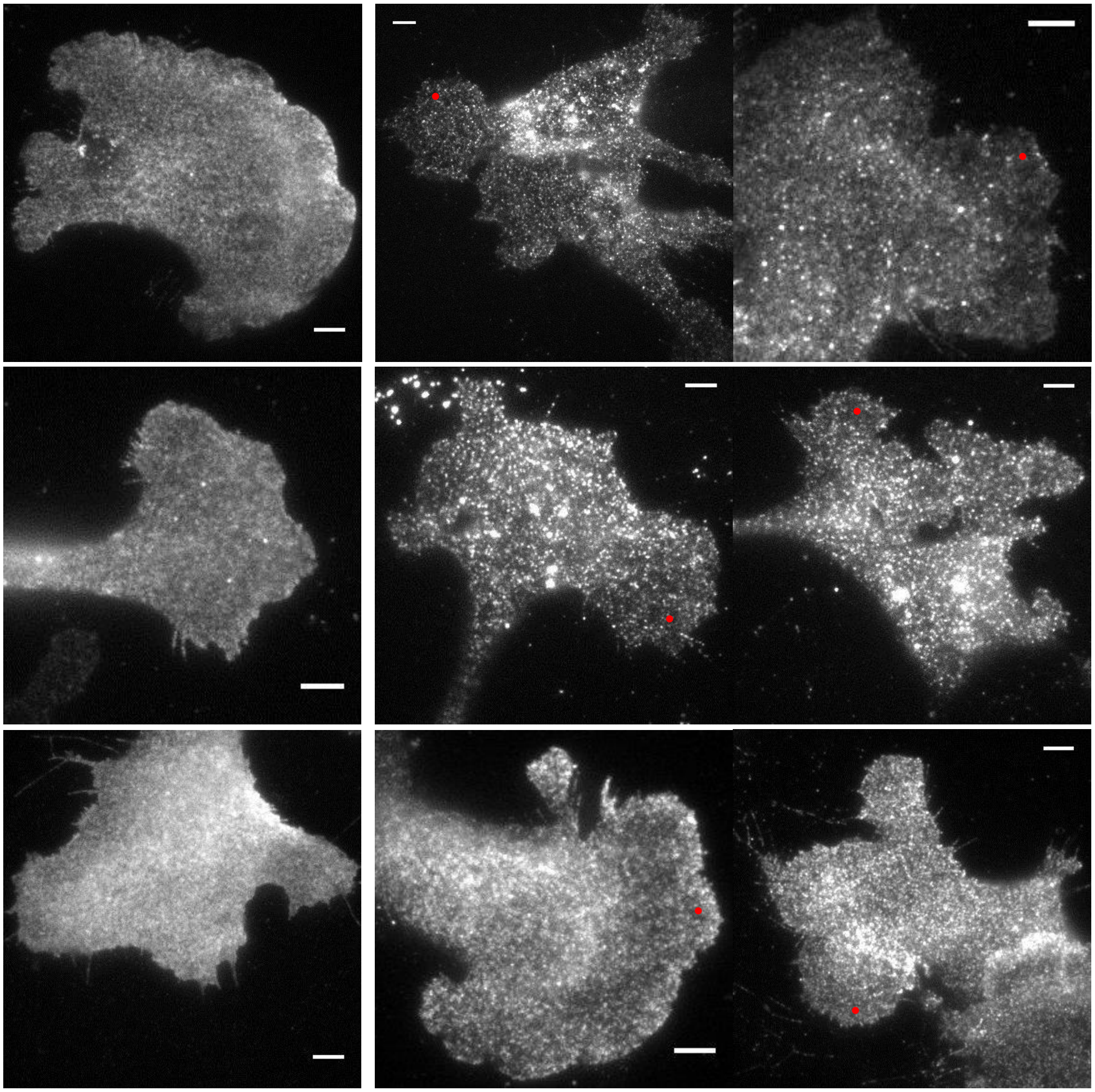

Figure S9. TIRF images of 728 cells with EphA2FL-GFP (upper row), EphA2ΔS-GFP (middle row) and EphA2ΔKS-GFP (lower row) for FCS measurements. Images in the first column represent 728 cells before ephrinA1-Fc treatment and images in the second and third columns represent 728 cells with ephrinA1-Fc treatment. Upon ephrinA1-Fc treatment, clusters formation can be observed. The positions of laser beam were marked as red dots. Laser beam was always focused at membrane area on the edge of the cell, avoiding any bright features. All scale bars are 5 μm.

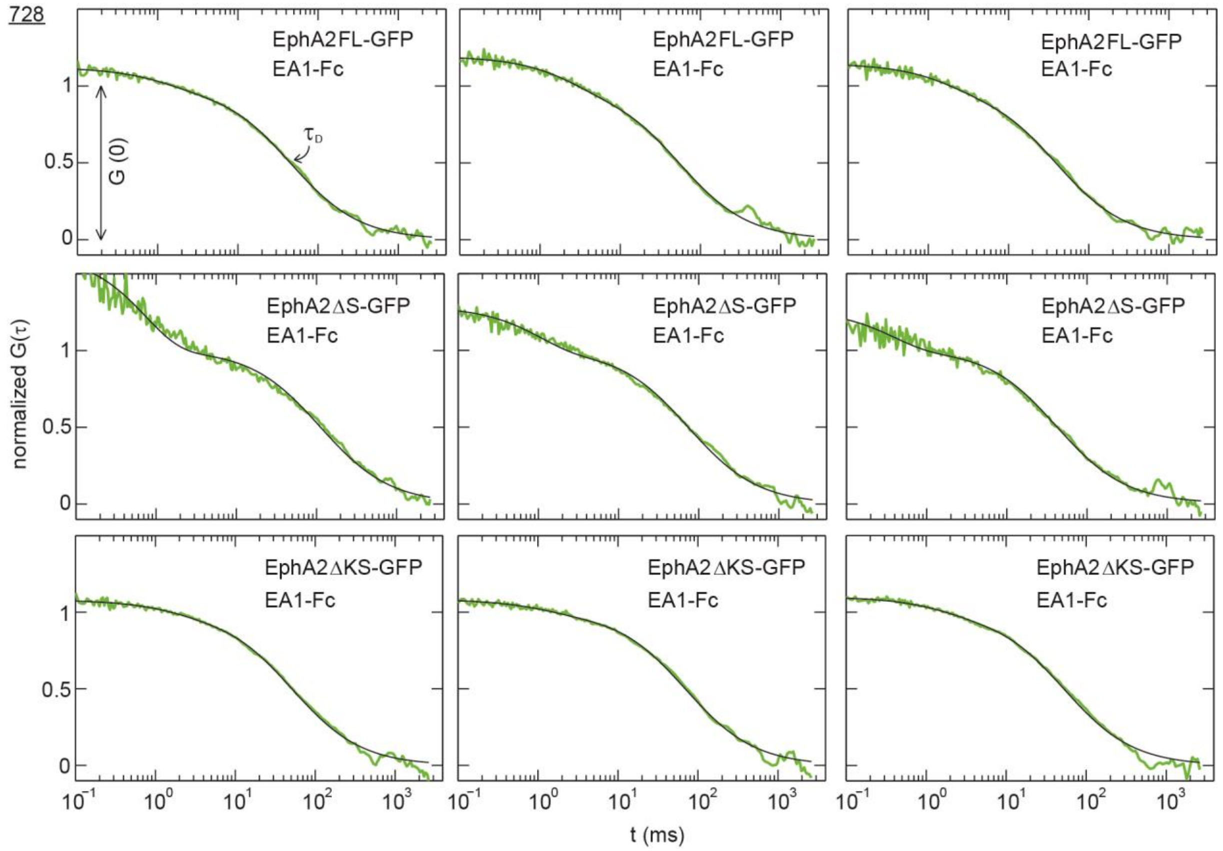

Figure S10. Representative auto-correlation curves of FCS measurements on ephrinA1-FC (EA1-Fc) treated 728 cells with expression of truncation mutant of EphA2 constructs. τ_D reports on mobility of the diffusive receptors and is used to calculate the diffusion coefficients. G (0) reports on concentration of the diffusive receptors and is used to calculate molecular brightness.

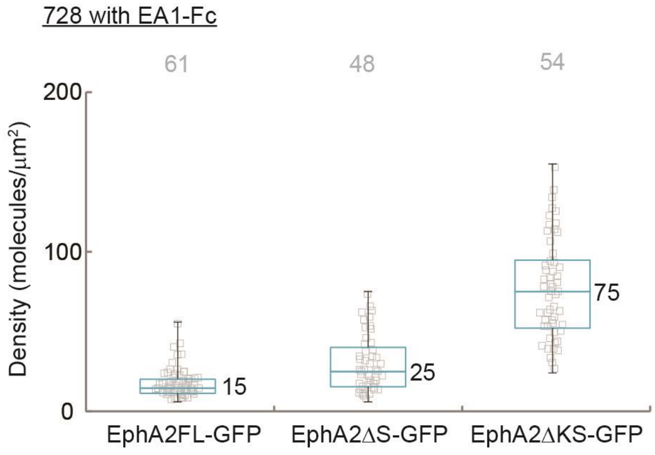

Figure S11. Density of truncation mutant constructs of EphA2 in 728 cells with ephrinA1-Fc treatment. The median values were reported next to the box plots. Each data point was the average of five 15s FCS measurements performed on one cell. The grey numbers on top the plots are the total number of cells used.

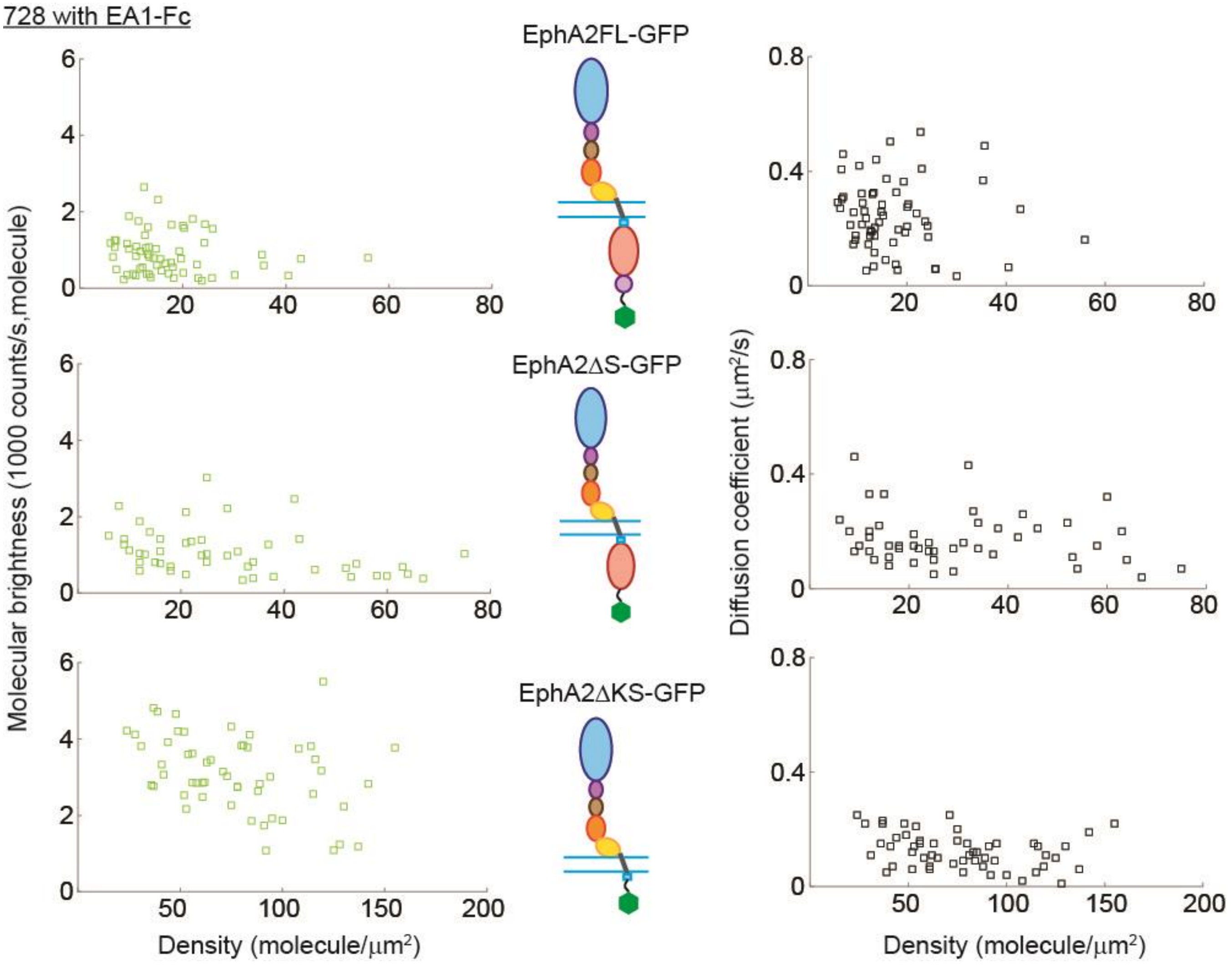

Figure S12. Molecular brightness (left, green) and diffusion coefficient (right, black) of truncation mutant constructs of EphA2 in 728 cells with ephrinA1-Fc treatment against receptor density. Each data point was the average of five 15s FCS measurements performed on one cell. There is no obvious dependence of molecular brightness or diffusion coefficient on receptor density.
